# Computed tomography angiography study of variations of the celiac trunk and hepatic artery in 100 patients

**DOI:** 10.1590/0100-3984.2016.0179

**Published:** 2018

**Authors:** Ivelise Regina Canito Brasil, Igor Farias de Araujo, Adriana Augusta Lopes de Araujo Lima, Ernesto Lima Araujo Melo, Ronaldo de Matos Esmeraldo

**Affiliations:** 1 PhD, Adjunct Professor of Clinical Surgery, School of Medicine, Universidade Estadual do Ceará (UECE), Head of the Liver Transplant Program at the Hospital Geral de Fortaleza (HGF), Fortaleza, CE, Brazil; 2 Medical Student at the Universidade Estadual do Ceará (UECE), Fortaleza, CE, Brazil; 3 PhD, Substitute Professor of Anatomy, School of Medicine, Universidade Estadual do Ceará (UECE), Fortaleza, CE, Brazil; 4 PhD, Adjunct Professor of Diagnostic Imaging, School of Medicine, Universidade Estadual do Ceará (UECE), Fortaleza, CE, Brazil; 5 MD, General Surgeon, Hospital Geral de Fortaleza (HGF), Fortaleza, CE, Brazil

**Keywords:** Anatomy, Computed tomography, Celiac artery, Hepatic artery, Liver transplantation

## Abstract

**Objective:**

To describe the main anatomical variations of the celiac trunk and the hepatic artery at their origins.

**Materials and Methods:**

This was a prospective analysis of 100 consecutive computed tomography angiography studies of the abdomen performed during a one-year period. The findings were stratified according to classification systems devised by Sureka et al. and Michels.

**Results:**

The celiac trunk was "normal" (i.e., the hepatogastrosplenic trunk and superior mesenteric artery originating separately from the abdominal aorta) in 43 patients. In our sample, we identified four types of variations of the celiac trunk. Regarding the hepatic artery, a normal anatomical pattern (i.e., the proper hepatic artery being a continuation of the common hepatic artery and bifurcating into the right and left hepatic arteries) was seen in 82 patients. We observed six types of variations of the hepatic artery.

**Conclusion:**

We found rates of variations of the hepatic artery that are different from those reported in the literature. Our findings underscore the need for proper knowledge and awareness of these anatomical variations, which can facilitate their recognition and inform decisions regarding the planning of surgical procedures, in order to avoid iatrogenic intraoperative injuries, which could lead to complications.

## INTRODUCTION

The trifurcation of the celiac trunk was first described by Haller in 1756. In 1955, Michels developed a system for classifying the anatomical pattern of the celiac trunk, based on the dissection of 200 cadavers. In 1966, an international classification system for anatomical variations of the hepatic artery was proposed^1-5^.

The celiac trunk and the superior mesenteric artery are two branches of the abdominal aorta. The celiac trunk emerges from just below the aortic hiatus at the level of the transition from the thoracic vertebrae to the lumbar vertebrae. In the so-called "normal" anatomical pattern, the celiac trunk trifurcates (into the splenic artery, common hepatic artery, and left gastric artery) slightly below the point at which it emerges from the aorta^4,6-11^.

It is now known that the abdominal vasculature has several common patterns of origin. Knowledge of the most common anatomical variations is crucial in surgical planning and in interventional examinations^6,10,12-19^.

With advances in the imaging techniques employed in angiography-computed tomography (CT) and magnetic resonance-not only in data acquisition but also in image post-processing on workstations, it is possible to obtain information that facilitates the planning of a given surgical procedure, which can, ultimately, contribute to reducing the associated rates of morbidity and mortality. For example, one important application that requires a detailed knowledge of the vascular anatomy is the infusion of chemotherapy via catheter for the treatment of unresectable malignant liver tumors. Laparoscopic surgery can be understood as a model of the importance of recognizing vascular variations in order to avoid iatrogenic complications, given that the surgical field is limited^11,13,14,16,18-22^.

As it evolved, CT began to allow the acquisition of a greater quantity of computerized images in a shorter period of time, through the use of scanners with multiple rows of submillimeter detectors. Thus, CT facilitated the acquisition of images of the standard abdominal vasculature and its variations, as an aid in emergency situations, such as gastrointestinal bleeding^11,13,16,18,20,21,23^.

The objective of the present study was to evaluate the patterns of anatomical variations of the celiac trunk and hepatic artery. To that end, we analyzed CT angiography examinations performed in multidetector scanners.

## MATERIALS AND METHODS

The study was approved by the Research Ethics Committee of the General Hospital of Fortaleza, located in the city of Fortaleza, in the state of Ceará, Brazil. All of the patients included in the study had received a physician referral for the examination, due to causes unrelated to the research, and it was therefore unnecessary to obtain written informed consent. We prospectively analyzed 100 consecutive CT angiography examinations of the abdomen performed at the General Hospital of Fortaleza between June 2013 and June 2014. We excluded patients with a history of abdominal surgery.

### CT examination

The examinations were performed on a 64-channel multislice CT scanner (Brilliance; Philips Healthcare, Eindhoven, The Netherlands). We acquired source images with a thickness of 0.6 mm, reconstructed with a thickness of 2 mm and an increment of 1 mm. The contrast medium used was iobitridol (300 mg/mL, Henetix 300; Guerbet Produtos Radiológicos, Rio de Janeiro, Brazil), which was administered intravenously by injection pump (OptiVantage; Mallinckrodt, Cincinnati, OH, USA), with an 18-20 gauge catheter for peripheral access in the arm, at a flow rate of 4 mL/s. None of the patients included in this study had a reaction to the use of contrast.

### Radiological interpretation

The imaging data obtained were archived electronically and restored on a workstation (Advantage Workstation 4.4; General Electric Healthcare, Milwaukee, WI, USA), after which they were reconstructed by multiplanar reconstruction, maximum intensity projection, and volume rendering. Those techniques were actively applied during the interpretation phase, the images being interpreted by a radiologist with 14 years of experience in abdominal and vascular imaging.

In this study, we analyzed the anatomy of the celiac trunk, as well as the origins of the common hepatic, splenic, left gastric, and superior mesenteric arteries. We also examined the origins of the right hepatic artery, the left hepatic artery, the gastroduodenal artery, and any accessory hepatic arteries.

## RESULTS

### Variations of the celiac trunk

The anatomy of the celiac trunk was categorized according to the classification system devised by Sureka et al.^3^, as detailed in Table 1. We identified the normal anatomical pattern-the hepatogastrosplenic trunk and superior mesenteric artery originating from the abdominal aorta-in 43% of the cases. We also identified five patterns of anatomical variations: a hepatosplenic trunk with the left gastric artery emerging 0.4-2.5 cm above the bifurcation of the celiac trunk and the superior mesenteric artery emerging from the abdominal aorta, in 47% of the cases; a gastrosplenic trunk with the common hepatic artery and superior mesenteric artery originating from the abdominal aorta, in 2%; a gastrosplenic trunk with the hepatic artery emerging from the superior mesenteric artery, in 3%; a hepatosplenic mesenteric trunk with the left gastric artery originating from the abdominal aorta, in 1%; and an ambiguous anatomical pattern, which did not meet the criteria for any of the other patterns, in 4%.

**Table 1:** Anatomical variation of the celiac trunk in 100 patients, according to the classification system devised by Sureka et al.^(^^3^^)^.

Anatomical pattern - celiac trunk	Number of patients
Normal anatomy (HGSpT + SMA)	43
Anatomical variations	57
HSpT + LGA + SMA	47
GSpT + CHA + SMA	2
GSpT + HMT	3
CMT	0
HMT + LGA + SpA	0
HSpMT + LGA	1
Ambiguous pattern	4

HGSpT, hepatogastrosplenic trunk; SMA, superior mesenteric artery; HSpT, hepatosplenic trunk; LGA, left gastric artery; GSpT, gastrosplenic trunk; CHA, common hepatic artery; HMT, hepatic mesenteric trunk; SpA, splenic artery; HSpMT, hepatosplenic mesenteric trunk; CMT, celiacomesenteric trunk.

### Variations of the hepatic artery

**Figure 1:**
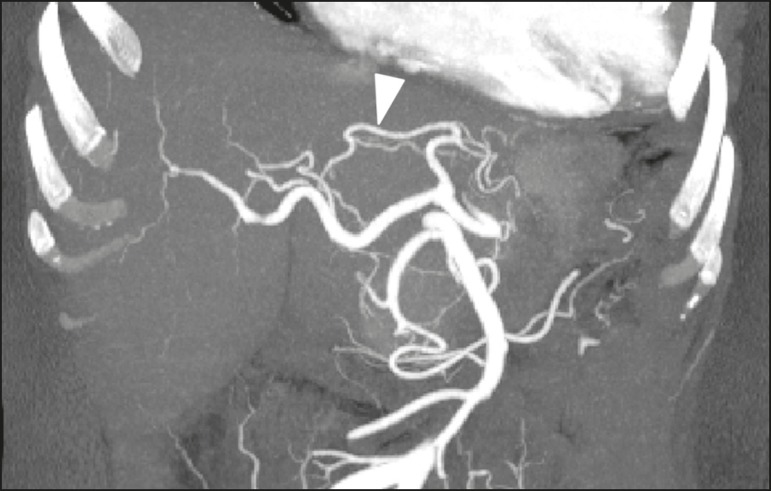
CT angiography reconstruction by maximum intensity projection showing the left hepatic artery (arrowhead) emerging from the left gastric artery.

**Figure 2:**
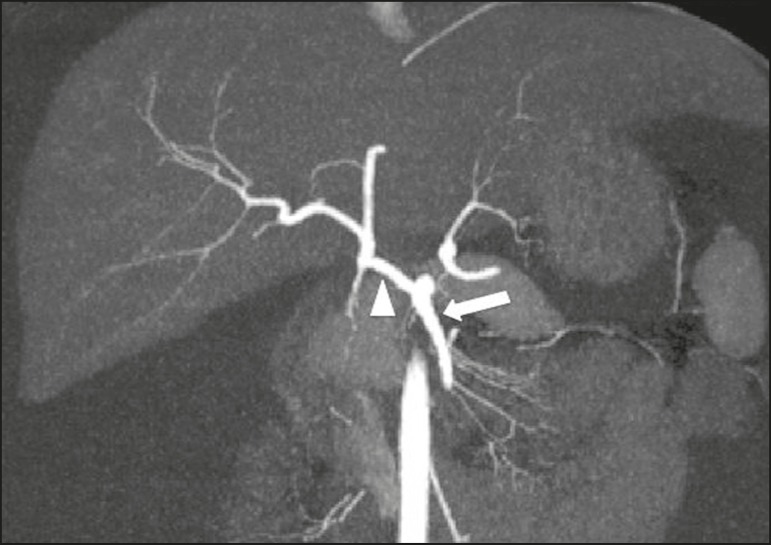
CT angiography reconstruction by maximum intensity projection in the coronal plane, showing the common hepatic artery (arrowhead) emerging from the superior mesenteric artery (arrow).

The anatomy of the hepatic artery was categorized according to the classification system devised by Michels^5^, as detailed in Table 2. We identified the normal anatomical pattern-the hepatic artery emerging from the common hepatic artery and bifurcating into the right and left hepatic arteries, designated type I-in 82% of the cases. We also identified six patterns of anatomical variations: the left hepatic artery emerging from the left gastric artery (type II), as illustrated in Figure 1, in 1% of the cases; the right hepatic artery emerging from the superior mesenteric artery (type III), in 10%; an accessory left hepatic artery emerging from the left gastric artery (type V), in 1%; an accessory right hepatic artery emerging from the superior mesenteric artery (type VI), in 1%; a common hepatic artery emerging from the superior mesenteric artery (type IX), as illustrated in Figure 2, in 4%; and an unspecified anatomical pattern (type XI), in 1%. None of the patients in our study showed the Michels type IV, VII, VIII, or X anatomical pattern.

**Table 2:** Anatomical variation of the hepatic artery in 100 patients, according to the classification system devised by Michels^(^^5^^)^.

Type	Number of patients	Description
I	82	PHA emerging from the CHA, bifurcating into the RHA and LHA
II	1	LHA emerging from the LGA
III	10	RHA emerging from the SMA
IV	0	RHA and LHA emerging from the LGA
V	1	Accessory LHA emerging from the LGA
VI	1	Accessory RHA emerging from the SMA
VII	0	Accessory RHA emerging from the SMA and acces-sory LHA emerging from the LGA
VIII	0	New origin of the RHA and of the accessory LHA or new origin of the LHA and of the accessory RHA
IX	4	CHA emerging from the SMA
X	0	CHA emerging from the LGA
XI	1	Variation not meeting the criteria for any of the other types (I through X)

PHA, proper hepatic artery; CHA, common hepatic artery; RHA, right hepatic artery; LHA, left hepatic artery; LGA, left gastric artery; SMA, superior mesenteric artery.

## DISCUSSION

We prospectively analyzed 100 consecutive CT angiography examinations of the abdomen performed at the General Hospital of Fortaleza over a one-year period. The imaging evaluation of the rhino-orbital region has been the object of a series of recent publications in the radiology literature of Brazil^24-29^.

In the standard definition of the visceral anatomy, the celiac trunk originates from the abdominal aorta and trifurcates into the left gastric artery, splenic artery, and common hepatic artery^4,7,30^. Although there are 15 possible patterns of anatomical variations of the celiac trunk, we detected, in our study, only six types, the same number found in the study conducted by Sureka et al.^3^. In our study, the normal anatomic pattern of the celiac trunk was found in 43% of the cases, compared with 89% in the dissection study conducted by Michels^5^; 91% in the study conducted by Sureka et al.^3^; 86% in the study conducted by Sankar et al.^8^; 85.1%, 89.5%, and 95.4%, respectively, in cadaver studies, imaging studies, and liver transplantation studies, as reported by Panagouli et al.^31^; 89.1% in the study conducted by Song et al.^18^; 89.8% in the study conducted by Chen et al.^32^, who analyzed a population defined as homogeneous in Japan; and 90% in the study conducted by Araujo-Neto et al.^33^. We identified variations of the celiac trunk in 57% of the patients in our sample, compared with the 10.6% reported by Panagouli et al.^31^, the 5.5% reported by Sureka et al.^3^, the 14% reported by Sankar et al.^8^, the 9.6% reported by Song et al.^18^ , and the 10.2% reported by Chen et al.^32^.

The most common anatomical pattern of variation in our study-found in 47% of the cases-was a hepatosplenic trunk with the left gastric artery emerging 0.4-2.5cm above the bifurcation of the celiac trunk and the superior mesenteric artery originating from the abdominal aorta. That anatomical pattern was also observed by Sureka et al.^3^, Michels^5^, and Song et al.^18^ , although in only 2.3%, 4%, and 4.42% of the cases, respectively. In the study conducted by Araujo-Neto et al.^33^, the most common anatomical pattern of variation-found in 8.3% of the cases-was a hepatosplenic trunk with the left gastric artery originating from the abdominal aorta.

Regarding the anatomy of the hepatic artery, the most commonly seen pattern, according to the classification system devised by Michels^5^, is type I; that is, the hepatic artery emerging from the common hepatic artery and bifurcating into the right and left hepatic arteries^5^. In our study, we found Michels type I anatomical pattern of the hepatic artery in 82% of the cases, whereas Gumus et al.^15^, Sureka et al.^3^, and Chen et al.^1^ reported Michels type I anatomical pattern in 66.8%, 55%, and 51%, respectively. In a cadaver study conducted in Brazil, Sebben et al.^2^ identified Michels type I anatomical pattern in 73% of the cadavers dissected. In a liver transplantation study conducted in the Brazilian state of Paraná, Freitas et al.^30^ found the normal anatomical pattern in 76.82% of the cases.

In our study, 18% of the patients had some variation in the anatomy of the hepatic artery, compared with 33.2% reported by Gumus et al.^15^, 45% reported by Sureka et al.^3^, 49% reported by Chen et al.^1^, 27% reported by Sebben et al.^2^, 23.18% reported by Freitas et al.^30^, 10.2% reported by Chen et al.^32^, and 21.7% reported by Araujo-Neto et al.^33^. The most common anatomical pattern of variation in our study-found in 10% of the cases-was the right hepatic artery emerging from the superior mesenteric artery. In the studies analyzed, that was also the main pattern of anatomical variation, although in different proportions of the samples evaluated. Gumus et al.^15^, reported that same pattern in 10.1% of the patients in their sample, comparable to the 11% reported by Sureka et al.^3^, the 15% reported by Chen et al.^1^, the 10% reported by Sebben et al.^2^, and the 11.38% reported by Freitas et al.^30^.

Our study provides an insight into the anatomical patterns found in Brazil. In view of our findings, it is possible to conclude that, because of the extensive miscegenation in Brazil, the indices of anatomical variation, despite sharing points of similarity, also present patterns of variations that differ considerably from those reported in the international literature, especially when we compare the patterns of celiac trunk variation with those found in homogeneous populations^2,9,14,32^.

It is noteworthy that some isolated studies conducted in Brazil have analyzed the anatomical pattern of the hepatic artery and its branches in cadavers and during liver transplantations. However, studies dealing with the anatomical variations of the celiac trunk are really quite rare in Brazil, this being the aspect of our study that distinguishes it from others in the literature^2,9,14,22,30^.

On the basis of our findings, we can state that studies like ours are scarce and should be encouraged, in order to improve understanding of the anatomical patterns in the population of Brazil. Further studies of this nature could lead to better technical planning of surgical procedures and could avoid inadvertent injuries that might compromise the results of medical procedures, leading to complications. Better knowledge of anatomical variations could ultimately contribute to reducing the rates of morbidity and mortality in endovascular procedures, abdominal surgeries, and transplantations, especially those of the liver and pancreas^2,14,22,30^.
